# Clinical characteristics and risk factors of *Aeromonas* bloodstream infections in patients with hematological diseases

**DOI:** 10.1186/s12879-022-07277-7

**Published:** 2022-03-29

**Authors:** Chunhui Xu, Qingsong Lin, Yuanqi Zhao, Guoqing Zhu, Erlie Jiang, Shangzhu Li, Yingchang Mi, Yizhou Zheng, Fengkui Zhang, Xiaofan Zhu, Zhijian Xiao, Mingzhe Han, Jianxiang Wang, Sizhou Feng

**Affiliations:** 1 Clinical Laboratory, State Key Laboratory of Experimental Hematology, Haihe Laboratory of Cell Ecosysterm, Institute of Hematology and Blood Diseases Hospital, National Clinical Research Center for Blood Diseases, Chinese Academy of Medical Sciences and Peking Union Medical College, Tianjin, 300020 China; 2 Hematopoietic Stem Cell Transplantation Center, State Key Laboratory of Experimental Hematology, Haihe Laboratory of Cell Ecosysterm, Institute of Hematology and Blood Diseases Hospital, National Clinical Research Center for Blood Diseases, Chinese Academy of Medical Sciences and Peking Union Medical College, Tianjin, China; 3 General Medical Center for Blood diseases, State Key Laboratory of Experimental Hematology, Haihe Laboratory of Cell Ecosysterm, Institute of Hematology and Blood Diseases Hospital, National Clinical Research Center for Blood Diseases, Chinese Academy of Medical Sciences and Peking Union Medical College, Tianjin, China; 4 Leukemia Center, State Key Laboratory of Experimental Hematology, Haihe Laboratory of Cell Ecosysterm, Institute of Hematology and Blood Diseases Hospital, National Clinical Research Center for Blood Diseases, Chinese Academy of Medical Sciences and Peking Union Medical College, Tianjin, China; 5 Anemia Center, State Key Laboratory of Experimental Hematology, Haihe Laboratory of Cell Ecosysterm, Institute of Hematology and Blood Diseases Hospital, National Clinical Research Center for Blood Diseases, Chinese Academy of Medical Sciences and Peking Union Medical College, Tianjin, China; 6 Pediatric Hematology Center, State Key Laboratory of Experimental Hematology, National Clinical Research Center for Blood Diseases, Haihe Laboratory of Cell Ecosysterm, Institute of Hematology and Blood Diseases Hospital, Chinese Academy of Medical Sciences and Peking Union Medical College, Tianjin, China; 7 Myelodysplastic Syndromes Center, State Key Laboratory of Experimental Hematology, National Clinical Research Center for Blood Diseases, Haihe Laboratory of Cell Ecosysterm, Institute of Hematology and Blood Diseases Hospital, Chinese Academy of Medical Sciences and Peking Union Medical College, Tianjin, China; 8 Hematopoietic Stem Cell Transplantation Center, State Key Laboratory of Experimental Hematology, Haihe Laboratory of Cell Ecosysterm, Institute of Hematology and Blood Diseases Hospital, National Clinical Research Center for Blood Diseases, Chinese Academy of Medical Sciences and Peking Union Medical College, No. 288 Nanjing Road, Tianjin, China

**Keywords:** *Aeromonas*, Bacteremia, Risk factors, Hematological diseases

## Abstract

**Background:**

To analyze the clinical features, risk factors and outcomes of *Aeromonas* bloodstream infections (BSIs) in patients with hematological diseases to establish an effective optimal therapy against it.

**Methods:**

A retrospective study was performed by reviewing medical records of patients admitted to a tertiary blood disease hospital in China. Patients with hematological diseases who suffered from *Aeromona*s bacteremia during January 2002 to December 2020 were enrolled in this study.

**Results:**

A total of 63 patients who developed *Aeromonas* bacteremia were enrolled in the study, and 91.9% of patients were neutropenic at the onset of BSIs. The major complications were skin and soft tissue infection (SSTI) (22.2%), followed by gastroenteritis (19.0%) and pneumonia (14.3%). High carbapenem resistance rates (70.8% for imipenem, 71.4% for meropenem) were note among the cases. Furthermore, *Aeromonas* strains isolated from five individuals developed resistance to quinolone, β-lactams and tigecycline during the therapy. The 30-day mortality rate was 15.9%, while bacteremia with SSTI showed a much worse prognosis, with 50.0% (7/14) of the patients dying within 30 days of initiating the therapy. In the multivariate analysis, SSTI (OR = 28.72; 95% CI, 1.50–551.30; *P* = 0.026) and shock (OR = 47.58; 95% CI,1.06–2126.80; *P* = 0.046) were independent risk factors for mortality.

**Conclusions:**

*Aeromonas* bacteremia usually occurred in patients with neutropenic status, and patients with SSTIs were more likely to show a worse prognosis. Carbapenems should be avoided in patients with *Aeromonas* BSIs and SSTIs given high resistance rate.

## Introduction

*Aeromonas* species are gram-negative, oxidase-positive, facultative anaerobic, distributed widely in the aquatic environment, including groundwater, water treatment systems, rivers, and lakes [1, 2]. *Aeromonas* can cause gastroenteritis disease [3], skin and soft tissue infection (SSTI) [4, 5], pneumonia, and bloodstream infections (BSIs) [6, 7]. *Aeromonas* infection can be life-threatening and invasive in immunocompromised individuals. The clinical characteristics and risk factors associated with mortality by *Aeromonas* vary significantly with population and region [8, 9]. BSIs are common among immunocompromised hosts, with the mortality being as high as 68% [10–12]. Carbapenem is commonly recommended in high-risk patients with febrile neutropenia because it has a broad-spectrum and activity against gram-positive and gram-negative bacteria. In contrast to other gram-negative bacilli, *Aeromonas* can express various chromosomal β-lactam-induced β-lactamases, resulting in carbapenem resistance and treatment failure [13, 14]. At present, there is a limited research focus on *Aeromonas* BSIs in patients with hematological diseases in China. In this study, we retrospectively analyzed the clinical characteristics of patients with blood diseases complicated with *Aeromonas* BSIs in the past 19 years. We investigated the antibiotic resistance profiles of isolated strains, which can provide infection prevention and treatment recommendations.

## Materials and methods

### Data collection

We retrospectively reviewed patients diagnosed with hematologic diseases and *Aeromonas* bacteremia from January 2002 to December 2020 at the Institute of Hematology & Blood Diseases Hospital, a 766-bed tertiary teaching hospital in Tianjin, China. Medical records, including demographic characteristics, clinical symptoms, antimicrobial susceptibility profiles, antibiotic treatment, and outcomes were recorded. A total of 63 patients with hematological disorders complicated with *Aeromonas* BSIs were identified and assessed. Each case was evaluated as clinically relevant by an experienced physician with reference to clinical and laboratory criteria. The local ethics committee approved the study.

### Strain identification and antimicrobial sensitivities

*Aeromonas* BSI was based on the diagnostic criteria as at least one blood culture being positive. Blood culture was performed by using an automatic blood culture system (BD, USA), and the isolated *Aeromonas* strains were identified with reference to the National Guide to Clinical Laboratory Procedures. VITEK 2 compact (bioMérieux, France) was used to identify the isolates and MALDI-TOF MS (bioMérieux, France) for further confirmation. VITEK 2 Compact AST GN67 and XN04 (bioMérieux, France) were used to conduct antimicrobial susceptibility tests by using an automated system. The Minimal inhibitory concentration (MIC) was measured according to the Clinical and Laboratory Standards Institute (CLSI) M45 document guidelines.

### Definitions

Neutropenia was defined as an absolute neutrophil count (ANC) below 0.5 × 10^9^/L, or the ANC is expected to decrease below 0.5 × 10^9^/L over the 48 h. Severe neutropenia was defined as the ANC below 0.1 × 10^9^/L. Unsolved neutropenia was defined as neutropenia lasting longer than 14 days or not resolved in 14 days after the onset of BSIs. *Aeromonas* bacteremia was defined as present when blood cultures were positive for *Aeromonas*. The date of blood culture collection was defined as the onset of BSI. Empirical treatment choices of antibiotics were defined as appropriate when they were sensitive in vitro in the first 24 h after BSIs. Definitive treatment was the choice of antibiotics after the susceptibility results were available. Previous antibiotic exposure was defined as at least 72 h of antibiotic use within 30 days before BSIs.

### Statistical analyses

For categorical variables, the Pearson’s Chi-square or Fisher’s exact test was applied. Quantitative data were expressed as medians with ranges, while qualitative data were expressed as a proportion ratio of at least three individuals. Correlational analyses was conducted by Spearman’s test of a cross contingency table. *P* < 0.05 was considered to indicate a statistically significant difference. Various variables were evaluated as potential risk factors for 30-day mortality in univariate and multivariate analyses by using a logistic regression model. SPSS software 24.0 (Statistical Product and Service Solutions, Chicago, IL, USA) was used to analyze the data.

## Results

### Clinical characteristics of the enrolled patients

A total of 63 patients (age: 4–63 years; median age: 31 years; 40 men, 23 women) were finally included in the study. The clinical characteristics of these patients are summarized in Table 1. Acute myeloid leukemia (AML) (34, 54.0%) was the most common disorder, followed by acute lymphoblastic leukemia (ALL) (17, 27.0%), severe aplastic anemia (SAA) (9, 14.3%), myelodysplastic syndromes (MDS) (2, 3.2%), and acute heterozygosis leukemia (AHL) (1, 1.6%). In patients with acute leukemia, 53.8% (28/52) were in complete remission. Of all patients, 48 received chemotherapy, 4 received allogeneic hematopoietic stem cell transplantation (allo-HSCT), and 7 received immunosuppression therapy. In total, 91.9% of the patients had neutropenia at the onset of BSIs. In these neutropenic patients, 78.9% (45/57) had severe neutropenia condition. The median duration of neutropenia was 6 days (2 ~ 39) after BSIs. Neutropenia was considered unresolved in 29% of the patients. Hospital-acquired bacteremia was recorded in 57 (90.5%) cases.

**Table 1:** Demographic and clinical characteristics of patients with *Aeromonas* bacteremia according to the overall survival statues

Characteristics	Total (n = 63)	Survival (n = 53)	Non-survival (n = 10)	*P* value
Median age (range)	31 (4–63)	33 (7–63)	27 (4–57)	0.809
Male	40 (63.5)	32 (60.4)	8 (80.0)	0.302
Underlying diseases				0.327
Acute myeloid leukemia	34 (54.0)	30 (56.6)	4 (40.0)	
Acute lymphoblastic leukemia	17 (27.0)	15 (28.3)	2 (20.0)	
Severe aplastic anemia	9 (14.3)	6 (11.3)	3 (30.0)	
Myelodysplastic syndromes	2 (3.2)	1 (1.9)	1 (10.0)	
Acute heterozygosis leukemia	1 (1.6)	1 (1.9)	0 (0.0)	
Status of acute leukemia				0.397
Complete remission	28 (53.8)	26 (56.5)	2 (33.3)	
Treatment				0.139
HSCT	4 (6.3)	4 (7.5)	0 (0.0)	
Chemotherapy	48 (76.2)	42 (79.2)	6 (60.0)	
Immunosuppressive therapy	7 (11.1)	5 (9.4)	2 (20.0)	
Neutropenic				
Neutropenia at the onset of BSI	57 (91.9)	47 (90.4)	10 (100.0)	0.582
Severe neutropenia at the onset of BSI	45 (72.6)	37 (71.2)	8 (80.0)	0.713
Length of days for neutropenia before BSI, median (range)	4 (0–21)	4 (0–21)	8 (0–16)	0.083
Length of days for neutropenia after BSI, median (range)	6 (2–39)	7 (2–39)	5.5 (2–22)	0.82
Unresolved neutropenic	18 (29.0)	9 (17.3)	9 (90.0)	**< 0.001**
Hospital-acquired	57 (90.5)	49 (92.5)	8 (80.0)	0.24
Clinical presentation				
Fever	63 (100.0)	53 (100.0)	10 (100.0)	–
Shock	8 (12.7)	1 (1.9)	7 (70.0)	**< 0.001**
Gastroenteritis	12 (19.0)	10 (18.9)	2 (20.0)	1
Skin and soft tissue infection	14 (22.2)	7 (13.2)	7 (70.0)	**0.001**
Mucositis	7(11.1)	5 (9.4)	2 (20.0)	0.306
Pneumonia	9 (14.3)	7 (13.2)	2 (20.0)	0.626
Antimicrobial exposure within 30 days	21 (33.3)	16 (30.2)	5 (50.0)	0.28

All patients had fever (> 38℃) at the time of positive blood culture collection. Regarding the accompanying clinical complications, 14 (22.2%) patients developed SSTIs, 12 (19.0%) patients had digestive tract symptoms, 9 (14.3%) patients had pneumonia, 8 (12.7%) patients had experienced shocks, and 7 (11.1%) patients had mucositis. Twenty-one patients had antibiotic exposure in the 30 days preceding *Aeromonas* bacteremia.

The clinical characteristics of patients with *Aeromonas* bacteremia were compared based on their clinical outcomes (Table 1). No significant differences were noted in the age, sex, underlying hematologic diseases, status of leukemia, treatment of hematological diseases, or antimicrobial exposure between survivor and non-survivor groups. Unresolved neutropenia after BSIs (90.0% vs. 17.3%, *P* < 0.001) was more common in non-survivor group, and the patients with combined shock or SSTI showed higher mortality rates (shock: 87.5% vs. 5.5%, *P* < 0.001; SSTI 50% vs. 6.1%, *P* < 0.001).

### Clinical characteristics and 30-day outcomes of patients with SSTI concomitant infections

**Fig. 1:**
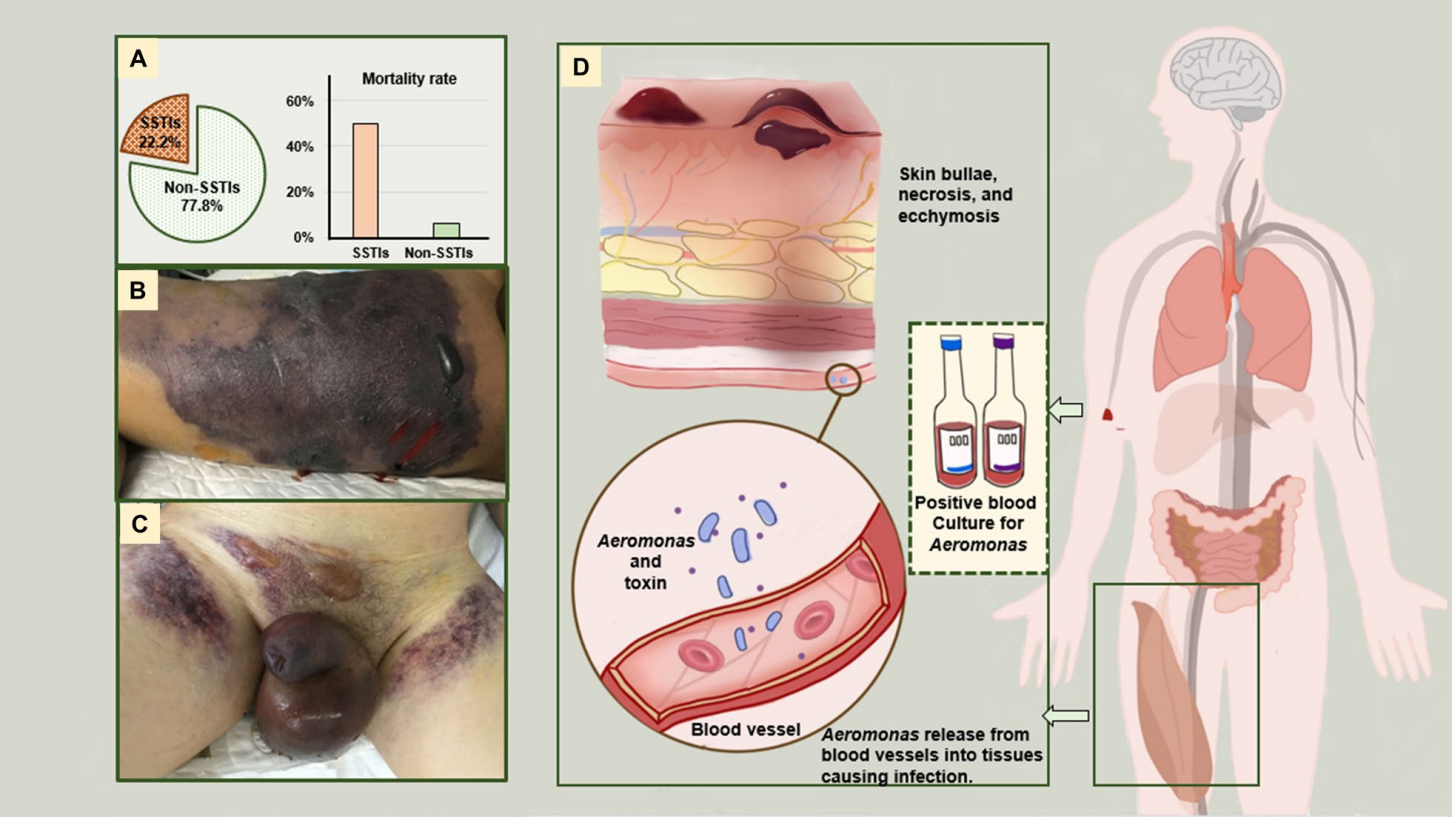
The clinical characteristics of patients with concomitant skin and soft tissue infections. **A** The ratio of patients who with SSTIs, and the mortality rates of patients with and without SSTIs. **B** The patients’ lower limbs showed swelling with massive subcutaneous bruising and blister formation. **C** The patient with bilateral swelling of the lower extremities and massive subcutaneous bruising with blister formation bilaterally around navel and groin. The perineum and scrotum were swollen with bruising. **D** Depiction of the possible mechanism by which *Aeromonas* causes skin and soft tissue infection through bloodstream infection. Necrotizing soft tissue infection often involves limbs, perineum, and abdomen. The lower extremities are most commonly involved and may present with erythema, severe pain, skin bullae, necrosis, or ecchymosis

Accompanying SSTI was recorded in 14 (22.2%) cases, which were cited as the most common case of involvement. None of the 14 patients had a history of trauma or surgery. The clinical characteristics of these patients are depicted in Table 2; Fig. 1. The 30-day mortality rate was 50% (7/14) in these patients. All patients were neutropenic at the onset of *Aeromonas* BSIs, among which 11 patients had severe neutropenia. Of the SSTI cases, seven patients had necrotizing infections; only one patient survived, albeit with an amputation. The main symptoms of necrotizing infection presentation were severe pain at the involved sites, swelling, erythematosus, crepitus and bullous skin lesions (Fig. 1A–C). The most common area of necrotizing soft tissue infection was the limbs, which involved seven patients. Other commonly affected regions included the groin, buttocks, perineum and abdomen. One patient developed rhabdomyolysis, which was characterized by muscle pain, hematuria and elevated creatine kinase, and the patient died in 48 h. In other cases, only muscle pain and swelling were recorded, and these patients survived. Among these SSTIs isolates, *A. hydrophila* was the most prevalent species, followed by *A. sobria.* The isolates were resistant to carbapenem in all cases, except one.

**Table 2:** The clinical characteristics and 30-day outcomes of patients with skin and soft tissue infections

	Sex	Age	Underlying condition	CR	ANC (10^9^/L)	Duration of neutropenia (Days)	Clinical presentation	Sites of involvement	Necrotizing SSTI	Shock	Pathogen	CR-*Aeromonas*	CA or HA	Empirical therapy	Appropriate initial empirical treatment	Outcomes
1	M	4	AA	NA	0.21	24	Persistent groin pain	Groin	N	N	*Aeromonas sobria*	Y	CA	CAZ, SMZ	N	Death
2	F	6	ALL	N	0.01	18	Serve pain, skin bullae and necrosis	Lower limbs and perineum	Y	Y	*Aeromonas sobria*	N	HA	MEM	Y	Death
3	M	21	MDS	N	0	10	Serve pain, muscle swelling and necrosis	Lower limbs	Y	N	*Aeromonas hydrophila*	Y	CA	IPM	N	Death
4	M	12	ALL	N	0.03	12	Serve pain, muscle swelling and necrosis	Lower limbs and buttocks	Y	N	*Aeromonas hydrophila*	Y	HA	MEM	N	Amputation
5	M	13	AA	NA	0	4	Red papules and firm nodules in skin with pain	The four limbs	N	N	*Aeromonas hydrophila*	Y	HA	SCF	Y	Survival
6	M	27	AML	CR	0.05	7	Unilateral lower limb pain and muscle swelling	Unilateral lower limb	N	N	*Aeromonas hydrophila*	Y	HA	IPM	N	Survival
7	M	27	AA	NA	0.07	15	Serve pain, ecchymoses, necrosis	Starts at the knees and ankles then spreads throughout the body	Y	Y	*Aeromonas sobria*	Y	HA	IPM	N	Death
8	M	51	ALL	N	0	10	Skin swelling, bullae formation and necrosis	Lower limbs, abdomen and perineum	Y	Y	*Aeromonas hydrophila*	Y	HA	MEM、IPM	N	Death
9	M	54	AA	NA	0.03	18	Rhabdomyolysis: muscle pain, weakness, and dark urine	Muscles and multiple organs	Y	Y	*Aeromonas hydrophila*	Y	HA	MEM	N	Death
10	M	63	AA	NA	0.28	24	Muscle pain and swelling	Lower limbs	N	N	*Aeromonas sobria*	Y	HA	IPM	N	Survival
11	M	30	AML	N	0.27	21	Muscle pain	Lower limbs	N	N	*Aeromonas hydrophila*	NA	HA	MEM	NA	Survival
12	M	30	ALL	N	0.04	22	Balanoposthitis	Genitals	N	N	*Aeromonas hydrophila*	NA	HA	MEM	NA	Survival
13	M	11	ALL	N	0.01	18	Subcutaneous abscess on the left abdomen	Abdomen	N	N	*Aeromonas hydrophila*	NA	HA	SCF, MEM	NA	Survival
14	M	27	AML	CR	0	41	Skin swelling, bullae formation and necrosis	The four limbs	Y	Y	*Aeromonas sobria*	NA	HA	IPM, TZP, CIP	NA	Death

*CR* complete remission, *ANC* absolute neutrophil count, *M* male, *F* female, *CA* community-acquired, *HA* hospital-acquired, *AML* acute myeloid leukemia, *ALL* acute lymphoblastic leukemia, *AA* aplastic anemia, *MDS* myelodysplastic syndromes, *CAZ* ceftazidime, *MEM* meropenem, *IPM* imipenem, *SMZ* timethoprim–sulphamethoxazole, *SCF* cefoperazone/sulbactam, *TZP* piperacillin/tazobactam, *CIP* ciprofloxacin, *NA* not applicable or detected, *CR-Aeromonas* carbapenem resistant-*Aeromonas*

### Antibiotic resistance and resistance transformation

Automated systems for antimicrobial susceptibility tests were conducted in 2014. The results of the 49 *Aeromonas* clinical isolates are shown in Table 3. The sensitivity of *Aeromonas* to cephalosporins, quinolones and aminoglycosides was above 90%, which was higher than that of beta-lactam/beta-lactamase inhibitor combination antimicrobials (piperacillin/tazobactam, 67.3%). In comparison with other β-lactams, the resistance rates of carbapenems were much higher (imipenem, 70.8%, and meropenem, 71.4%). Remarkably, 12 *Aeromonas* strains isolated from 5 individuals changed from susceptible to resistant for different antimicrobial agents during the treatment (Table 4). The time interval between susceptible to resistant was 2, 6, 7, 8 and 19 days, respectively. The types of antibiotics that had resistance transformation included quinolones, carbapenems, cephalosporins, piperacillin/tazobactam and tigecycline. Most of the drugs with antimicrobial resistance transformation had a history of exposure to such types of antimicrobial agents. Quinolones were the most common drugs involved in resistance transformation, as noted in 4 of the patients.

**Table 3:** In vitro susceptibilities of 49 clinical isolates of *Aeromonas* spp

Antimicrobial agent	Resistant rate (n)	Breakpoints	
		S	R
Aztreonam	8.2 (49)	≤ 4	≥ 16
Cefuroxime	8.2 (49)	≤ 8	≥ 32
Ceftriaxone	8.2 (49)	≤ 1	≥ 4
Ceftazidime	6.1 (49)	≤ 4	≥ 16
Cefepime	0.0 (49)	≤ 8	≥ 32
Piperacillin/tazobactam	32.7 (49)	≤ 16/4	≥ 128 /4
Meropenem	71.4 (49)	≤ 1	≥ 4
Imipenem	70.8 (48)	≤ 1	≥ 4
Ciprofloxacin	4.1 (49)	≤ 1	≥ 4
Levofoxacin	2.1 (48)	≤ 2	≥ 8
Gentamicin	4.1 (49)	≤ 4	≥ 16
Amikacin	0.0 (49)	≤ 16	≥ 64
Tegacycline	0.0 (40)	≤ 2	≥ 8
Trimethoprim/Sulfamethoxazole	22.5 (49)	≤ 2/38	≥ 4/76

**Table 4:** Resistance transformation in 5 patients and the outcomes

	Pathogen	Antibiotic treatment	Time interval	MICs (the 1st isolate-the final isolate)								Outcomes
				FEP	TZP	MEM	IPM	CIP	LVX	GEN	TGC	
Case 1	*Aeromonas sobria*	CAZ, ET, SMZ, TGC, **FEP**, IPM, **CIP**	19	**< 1–64**	< 4	> 16	< 1	**1–>4**	**1–>8**	> 16	NA	Death
Case 2	*Aeromonas sobria*	IPM, AK, SCF, **MEM**	2	< 1	**< 4–128**	**< 0.25–>16**	< 1–4	1	1	2	NA	Survival
Case 3	*Aeromonas hydrophila*	SCF, **CIP**, IPM, ET	7	< 1	> 128	> 16	> 16	**1–>4**	1	< 1	1	Survival
Case 4	*Aeromonas hydrophila*	MEM, ET, **LVX**, CRO	8	< 1	> 128	> 16	8	**1–>4**	**1–>8**	< 1	**1–>8**	Amputation
Case 5	*Aeromonas sobria*	MEM, **TGC**, SCF	6	2	> 128	> 16	> 16	> 4	**2–>8**	< 1	**1–>8**	Survival

Bold format, the resistant transformation antibiotics

*MEM* meropenem, *IPM* imipenem, *CAZ* ceftazidime, *ET* etimicin, *SMZ* thimethoprim-sulphamethoxazole, *TGC* tegacycline, *SCF* cefoperazone/sulbactam, *CIP* ciprofloxacin, *LVX* levofloxacin, *FEP* cefepime, *CRO* ceftriaxone, *AK* amikacin, *GEN*,gentamicin, *NA* not detected

### Time distribution of infection

**Fig. 2:**
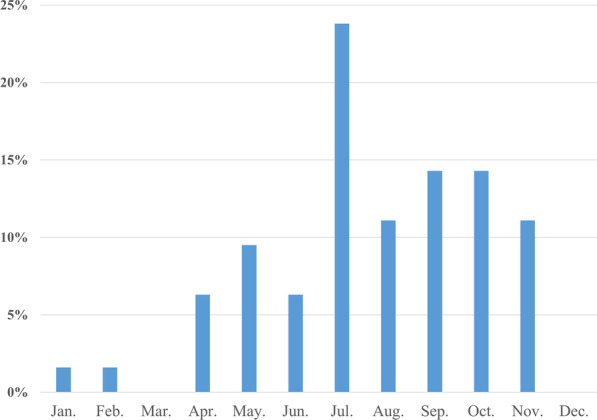
Monthly distribution of Aeromonas bacteremia

The detection times of *Aeromonas* BSI were recorded from 2002 to 2020 (Fig. 2). We observed a trend of greater frequency occurring between July and November (n = 47,74.6%) with the least frequent occurring between December and March (n = 2, 3.2%).

### Outcome analysis for non-survivors

A total of 10 patients included in the study died within 30 days of starting the treatment, resulting in an overall mortality rate of 15.9% (10/63). The median time between the occurrence of *Aeromonas* BSI and death was 6.5 days (range: 2–26). We analyzed the factors associated with 30-day overall case fatality after the onset of *Aeromonas* BSI, including unresolved neutropenia, SSTI, and shock by applying univariate analyses. In multivariate analysis, SSTI (OR = 28.72; 95% CI, 1.50–551.30; *P* = 0.026) and shock (OR = 47.58; 95% CI, 1.06–2126.80; *P* = 0.046) were independent risk factors associated with mortality during the 30-day follow-up (Table 5).

**Table 5:** Logistic regression modeling evaluating risk factors for mortality in patients with *Aeromonas* BSIs

Item	Univariate logistic regression			Multivariate logistic regression		
	OR	95% CI	*P* value	OR	95% CI	P value
Male	2.348	0.423–13.047	0.329	/	/	/
Age	0.999	0.958–1.042	0.96	/	/	/
ANC at the onset of BSI	6.414	0.019-2130.026	0.53	/	/	/
**Unresolved neutropenic**	**21.7**	**2.373–198.447**	**0.006**	14.003	0.799-245.273	0.071
**Shock**	**66.667**	**5.772–770.001**	**0.001**	**47.575 **	**1.064-2126.803**	**0.046**
Gastroenteritis	0.833	0.086–8.044	0.875	/	/	/
**Skin and soft tissue infection**	**27.75**	**4.134–186.274**	**0.001**	**28.716**	**1.496–551.300**	**0.026**
Appropriate empirical treatment	0.192	0.034–1.076	0.061	/	/	/
Antimicrobial exposure	2.727	0.580-12.831	0.204	/	/	/

**Fig. 3:**
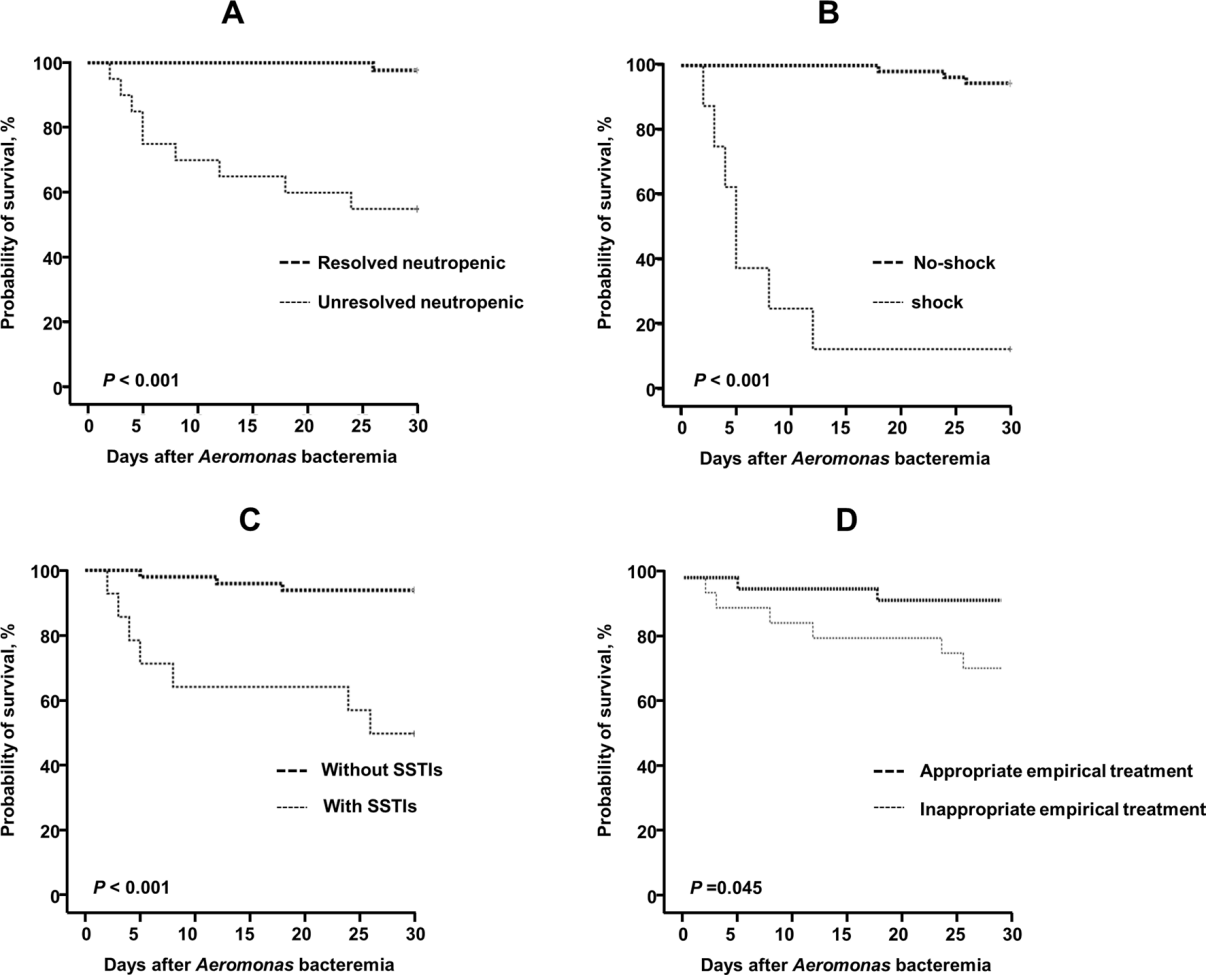
Kaplan-Meier curves of the 30-day probability of survival for BSI caused by *Aeromonas.* **A** Patients with and without unresolved neutropenic. **B** Patients with and without shock. **C** Patients with and without SSTI. **D** Patients with appropriate empirical treatment in 24 h

Kaplan–Meier survival analysis revealed that the survival of patients with unresolved neutropenia was significantly worse than that of patients with neutropenia resolved in 14 days (55.0% vs.97.6%, *P* < 0.001) (Fig. 3A). In addition, the 30-day death rate of patients who experienced shock was higher than that of patients without shock (87.5% vs. 5.8%, *P* < 0.001) (Fig. 3B), and patients with SSTIs or inappropriate treatment were associated with worse survival (SSTIs: 50.0% vs. 6.1%, *P* < 0.001; Inappropriate empirical treatment:28.6% vs. 7.1%, *P* = 0.045 ) (Fig. 3C, D).

## Discussion

Our study involved 63 cases of *Aeromonas* bacteremia. To the best of our knowledge, this is the largest single-center study until date in patients with hematological diseases. We retrospectively reviewed the laboratory and clinical characteristics as well as the treatment outcomes.

Over 19 years. *Aeromonas* BSIs demonstrated high mortality in patients with accompanying complications of SSTI or shock. Furthermore, we discovered that *Aeromonas* isolates were more resistant to carbapenem than other β-lactam antibiotics. These results together provided useful information for the better treatment of *Aeromonas* bacteremia.

Patients with hematological diseases are at a high risk of infection because of their disease features and receiving HSCT or chemotherapy [15]. Cases included in this study mainly consisted of acute leukemia patients (82.6%) and SAA patients (14.3%), and over 90% of *Aeromonas* BSIs occurred during the neutropenic stage. Neutropenia was not resolved in two weeks in 29.0% of the cases. Although underlying diseases were common in *Aeromonas* BSIs, such as solid tumors, liver cirrhosis, and hematological malignancies [16, 17], few previous studies have focused on *Aeromonas* BSIs in patients with hematological diseases [16, 18]. Limited information is available on antibiotic resistance in hospital-acquired infections.

All of the isolated were clinically relevant by clinical and laboratory criteria, and the following factors were taken into account: all the patients had fever at the time of the blood culture; trained nurses collected blood culture specimens by venipuncture of peripheral at the bedside; disinfecting steps had been properly followed in the blood collection with 2% chlorhexidine gluconate at the venipuncture site. Reller and colleagues showed that patients with neutropenia were significantly more likely to have a positive blood culture representing true bacteremia [19]; 91.9% of patients were neutropenic at the onset of BSIs.

In our study, most infections (90.5%) were hospital-acquired. Approximately 50% of the patients had only fever, and their clinical presentation of *Aeromonas* bacteremia was not specific, which can be challenging to clinicians. SSTIs are common complications, but their symptoms vary across patients. Although local inoculation can be a source of the SSTI in immunocompetent subjects [20, 21], none of the patients in our study had previous trauma or surgery. Moreover, signs and symptoms of SSTIs occurred at the same time as BSIs or later than BSIs, which suggests that the infection could be secondary to BSIs in most patients infected with *Aeromonas* rather than the source of BSIs (Fig. 1D). The mortality rate of patients with SSTIs was significantly higher (50.0%) than that of patients without SSTIs in this study. One surviving patient underwent amputation due to *Aeromonas* infection. Since *Aeromonas* can cause rapidly fatal infection in these patients, it should be considered an important pathogen for patients with hematological diseases when they have BSIs accompanied by complications of the SSTI. *Aeromonas* can cause mild to severe SSTIs [5, 22, 23]. The presentation includes cellulitis, myonecrosis, rhabdomyolysis and clostridium like gangrenous [24–26]. Although rhabdomyolysis is rarely described in patients with *Aeromonas* bacteremia, it is always fatal [17, 27]. In this study, one patient developed rhabdomyolysis and then rapidly developed secondary renal failure developed rapidly and died within 48 h.

Moreover, Schelenz’s study confirmed that patients with hematological disorders had a higher risk of BSIs than those with other cancers [28]. Carbapenem antibiotics are one of the most important empiric therapy for patients with hematological diseases as they exhibit a broad spectrum activity against gram-negative bacilli [29]. Unfortunately, after using carbapenems for a short time, carbapenem resistance emerges, including *Aeromonas* [30]. However, *Aeromonas* can exhibit rare phenotypes such as carbapenem resistance and cephalosporin susceptible. In this study, the resistance rate of *Aeromonas* to cephalosporins was less than 10%, while the resistance rate of carbapenems widely used in patients with hematological diseases was as high as 70%. This unusual resistance phenotype was related to its drug resistance mechanism, in which *Aeromonas* can produce b-metallo-β-lactamase (MBL) *cphA*, which is not commonly expressed but can be inducible and hydrolyze carbapenems [13, 31, 32]. More than 75% of *Aeromonas* carry *cphA* [13]. Meanwhile, the special mechanism of carbapenem resistance also develops false sensitivity in disk diffusion and E-test susceptibility testing [13]. Based on the findings in our study, caution use of carbapenem in patients with typical necrotizing SSTI symptoms accompanying BSIs is warranted.

In this study, the susceptible rate of *Aeromonas* to aminoglycosides and quinolones was > 90%, which is similar to the results of other research results [10, 33]. In conformance to other studies, it is worth noting that resistance induction was also found during the treatment period in five cases [30, 34]. Four patients developed quinolones resistance. One patient developed carbapenems, or piperacillin/tazobactam or tigecycline resistance. In three patients, the occurrences of antibiotic resistance were seen in two different types of antibiotics, which was the  first reported. This is an important clinical finding suggesting that monitoring blood cultures and antibiotic susceptibilities should be considered during a treatment process, especially among patients with immunocompromised conditions.

A total of 10 patients died within 30 days, presenting with an overall mortality rate of 15.9% in this study, which was lower than that reported previously [8, 16, 18, 35]. This finding could be associated with the common use of combination antibiotics in our patients with hematological diseases [36]. *Aeromonas* BSI was mainly detected at found in the neutropenia stage, and unresolved neutropenia was has been associated with high mortality. The symptoms varied among patients, with some patients reporting only fever before the positive results of blood culture, presented with fever of unknown origin, and the survival rate was high. Moreover, the mortality rate of patients receiving appropriate treatment demonstrated lower rate than that of patients who did not (Fig. 3D). Since only 10 patients had fatal outcomes, no significant correlation was recorded between appropriate treatment and outcomes. Our study found that SSTI and shock were independent risk factors for *Aeromonas* BSI mortalities. However, there were several limitations in our study. First, it was a retrospective and single-center study involving a small study group; therefore, the results may not be a true representative of other subject populations. Second, we used the VITEK auto system to perform susceptibility tests, this type of phenotypic method could fail to detect carbapenem resistance[13]. Third, since we didn’t preserve these isolates strains, molecular identification was not done in our study. Conventional microbiological identification methods had limitations in the accurate identification of *Aeromonas* to the species level.

## Data Availability

The datasets generated and/or analyzed during the current study are not publicly available considering the privacy or ethical restrictions, but are available from the corresponding author on a reasonable request.
